# Sunitinib Reduced the Migration of Ectopic Endometrial Cells via p-VEGFR-PI3K-AKT-YBX1-Snail Signaling Pathway

**DOI:** 10.1155/2022/6042518

**Published:** 2022-06-30

**Authors:** Xiaodan Fan, Yanyan Tong, Yiting Chen, Yichen Chen

**Affiliations:** ^1^Ningbo Institute of Medical Science, Zhejiang, China; ^2^Ningbo University, Zhejiang, China; ^3^Ningbo Women and Children's Hospital, Zhejiang, China

## Abstract

Endometriosis (EMs) is one of the most common gynecological diseases, lacking effective treatment. EMs are currently being treated with small molecule targeted therapy, which has resulted in a significant reduction in patient suffering. Our previous studies have shown that sunitinib plays an obvious role in migration. Consequently, the purpose of this study is to explore the molecular mechanism by which sunitinib suppressed the ectopic endometrial migration. The ectopic endometrial cells from patients were divided into two groups: the control group and the sunitinib group. Co-IP and protein spectrum assay were employed to filtrate differential proteins between two groups, and then, our study discovered a signaling pathway, p-VEGFR-PI3K-AKT-YBX1-Snail, in the cell of EMs. To confirm this signaling pathway, VEGF165 was added to the sunitinib group to upregulate the expression of VEGFR. Next, the expression of p-VEGFR, PI3K, AKT, YBX1, and snail was measured in the control group and sunitinib group (compared with the control group: p-VEGFR, PI3K, AKT, YBX1, and snail, ∗∗∗∗*P* < 0.0001) and the VEGFR+sunitinib group (compared with the sunitinib group: p-VEGFR, PI3K, AKT, and snail, ∗∗∗∗*P* < 0.0001; YBX1, ∗∗∗*P* < 0.001); finally, the outcome was as expected. In addition to in vitro experiments, we also conducted in vivo experiments in mice. In the EMs mouse model, we found sunitinib reduced the number of heterotopic foci (*t* = 11.16, ∗∗∗∗*P* < 0.0001) and inhibited the expression of p-VEGFR, YBX1, and snail by immunofluorescence. To sum up, sunitinib exactly reduced the migration of ectopic endometrial cells with the involvement of the p-VEGFR-PI3K-AKT-YBX1-Snail signaling pathway in both in vitro and in vivo experiments. This study suggests that sunitinib presents a potential targeted drug for EMs therapy.

## 1. Introduction

Endometriosis (EMs) is a common gynecological disease, affecting about 10% of women of reproductive age [1]. The major clinical manifestations are dysmenorrhea, abnormal menstruation, and infertility. Currently, the main clinical treatment methods include hormone therapy and surgical treatment. However, long-term hormone therapy, on the other hand, is being phased out as the female pregnancy rate declines. Meanwhile, surgery can cause patients to lose their fertility, which is harmful to women of reproductive age [2, 3]. Therefore, looking for a more appropriate targeted treatment of EMs has become a pressing matter.

Endometriosis is a chronic and inflammatory condition in which the endometrium appears outside of the uterus, most commonly in the ovaries, ligaments, and peritoneum [4]. Bartley et al. found that the expression of epithelial-mesenchymal transition- (EMT-) related proteins such as N-cadherin, twist, and snail in EMs was higher than that in normal endometrium [5]. In general, the rise of EMT is associated with increased cell migration and decreasing cell adhesion, leading to the promotion of tumor progression [6, 7]. Therefore, we considered that the signaling pathway within EMT-related proteins might play a vital role in the migration of ectopic endometrial cells.

Small molecule targeted therapy is a hot topic in clinical trials right now. Sophy, sunitinib, and ecotinib are examples of molecular targeted drugs that have emerged in recent years. However, most of these drugs are used in cancer treatment rather than in the treatment of EMs [8, 9]. Our team had screened several common targeted drugs on EMs in the previous research and found that sunitinib significantly inhibited the migration of cells. Sunitinib is a multitarget tyrosine kinase inhibitor, which targets VEGFR 1-3, PDGFR-*β*, and RET, taking antiangiogenic and antitumor effects [10–13]. Some studies have treated EMs with sunitinib in vivo and found that sunitinib indeed had an inhibitory effect on the heterotopic foci. However, the mechanism of sunitinib in the treatment of EMs is still unclear [14]. Therefore, our research team focused on the molecular mechanism of sunitinib inhibiting the migration of ectopic endometrial cells.

## 2. Materials and Methods

### 2.1. Isolation and Culture of Primary Endometrial Cells

Tissue samples were collected from 3 patients with endometriosis and 3 patients without endometriosis (details in STable 1). All enrolled patients did not receive hormone treatment in the 6 months. This study was approved by the Ethics Committee of Ningbo Women and Children's Hospital (Approval No: EC2019-037) in Zhejiang, China, and written informed consent was obtained. Fresh endometrial tissue samples of patients at Ningbo Women and Children's Hospital were obtained under sterile conditions. The tissue samples were digested with 1 mg/ml collagenase at 37°C for 1 hour. The supernatant was filtered through a 100 *μ*m filter screen. Finally, the cells which we collected above were cultured in Dulbecco's modified Eagle's medium (DMEM)/F12 supplemented with fetal bovine serum (FBS) at 37°C, 5% CO_2_. The P0-P1 generation was used in the following research.

Cells were divided into two groups after 80% of the medium had been covered: a control group and an experimental group. The experimental group was treated with 0.1 *μ*m sunitinib. After 24 hours, the following research was implemented.

### 2.2. Immunofluorescence Staining

Formalin-fixed paraffin-embedded (FFPF) adenomyosis tissues were sectioned into 4 *μ*M thick tissue slices. Slices were deparaffinized in xylene, rehydrated through graded ethanol, and boiled for 10 min in citrate buffer (pH 6.0) for antigen retrieval. Endogenous peroxidase activity was suppressed by exposure to 3% hydrogen peroxide for 10 min RT. Tissue sections were then permeabilized by 0.1% Triton X-100 (Sigma-Aldrich) for 10 min. Unspecific bindings were blocked by using PBS+5% normal goat serum for 1 h at RT. Tissue sections were incubated overnight at 4°C with the following anti-CD10 (1 : 50, Affinity, United States) and antivimentin (1 : 50, Affinity, United States). On the second day, slides were washed 3 times in PBS and incubated with the secondary antibody: goat anti-rabbit antibody 488 (1 : 500, Abcam, United States) for 1 h at RT in dark. Finally, sections were mounted using fluorescent mounting medium and visualized under the fluorescent microscope (Leica, Germany)

### 2.3. Transwell Insert Migration Assay

The control and experimental groups were digested and centrifuged, followed by cell resuspension in a serum-free medium. Then, the cell number was implanted to about 4 × 10^5^ cells/ml and added the cells to the upper compartment of the transwell chamber. F12 medium containing 10% FBS was placed in the lower compartment. After culturing for 24 h, it was fixed with 4% paraformaldehyde for 15 min and washed with PBS. After the transwell drying naturally, it was stained with crystal violet for 1 h and then washed. After air drying, a microscope selected 12 images at random and calculated the number of transmembrane cells.

### 2.4. VEGFR Agonist

The sunitinib group was treated with VEGF165 (MedChemExpress, New Jersey, USA), and the final concentration of VEGF165 was 20 nM. After 24 h incubation, the expression of related proteins among the control group, sunitinib treatment group, and sunitinib+VEGF165 treatment group was detected by Western blot.

### 2.5. Western Blot

The total cells were lysed with RIPA buffer supplemented with proteinase inhibitors. The protein concentrations were measured using the BCA protein assay kit. Protein samples were separated on a 10% SDS-PAGE electrophoresis gel and then transferred onto polyvinylidene fluoride (PVDF) membranes. The membranes were then blocked in skim milk solution for 1 hour at room temperature and then incubated overnight with primary antibodies at 4°C. The following primary antibodies were used in this blotting: anti-P-VEGFR (PA5-99362, Thermo Fisher Scientific, America), anti-VEGFR (ab36844, 1 : 1000, Abcam, Cambridge, UK), anti-snail (3879, 1 : 1000, Cell Signaling Technology, Boston, America), and anti-EMT Antibody Sampler Kit (9782, 1 : 1000, Cell Signaling Technology, Boston, America). The next day, after washing the membranes with TBST, the membranes were incubated with appropriate horseradish peroxidase- (HRP-) labeled secondary antibodies at room temperature for 1 h. Finally, the membranes were washed before being analyzed using an electrophoresis gel imaging system.

### 2.6. Immunoprecipitation

According to the Pierce MS-Compatible Magnetic IP Kit (Protein A/G) (20164, Thermo Fisher Scientific, Massachusetts, America) instructions, the following experiments were performed. Each group of proteins was mixed with a 5 *μ*l snail antibody (3879, 1 : 50, Cell Signaling Technology, Boston, America) and 500 *μ*l IP-MS Cell Lysis Buffer and incubated overnight at 4°C. The mixtures were added with 25 *μ*l prewashed magnetic beads and incubated at room temperature for 1 hour with mixing. Following that, all antibody subtypes were washed three times with buffer A and buffer B, followed by the addition of elution buffer to collect the magnetic beads and obtain precipitated proteins. Finally, the precipitated proteins can be processed for analysis using mass spectrometry.

### 2.7. Protein Identification via Mass Spectrometry

Firstly, the protein in the eluent was precipitated by the acetone method. After being washed with acetone and dried, the protein precipitation was treated with 8 M urea/100 mM Tris-HCl (pH 8.0) to fully dissolve the protein. Then, the protein was added with dithiothreitol (DTT) for 1 hour at 37°C. After that, the protein was alkylated with iodoacetamide (IAA) to block the sulfhydryl group. Later, the protein was treated with an appropriate volume of 100 mM Tris-HCl (pH 8.0), and the protein concentrations were quantified by the Bradford method. Then, the protein was incubated with trypsin at 37°C overnight for digestion. TFA was added the next day to finish the enzyme digestion, and the C18 column was used to desalinate the water. After being drained by the centrifugal concentrator, the product is stored at -20°C, waiting for the test.

Mass spectrometry was performed using the TripleTOF 5600 Liquid Chromatography-Mass (LC-MS) system of SCIEX company. The peptide sample was inhaled through the autosampler, then combined to the C18 capture column (5 *μ*m, 5 × 0.3 mm) and then eluted to the analytical column (75 *μ*m × 150 mm, 3 *μ*m particle size, 100 Å pore size, Eksigent) for separation. Then, a 30-minute analytical gradient was established by two mobile phases (mobile phase A: H2O, 0.1% formic acid; mobile phase B: ACN, 0.1% formic acid). The condition of MS/MS acquisition was that the parent ion signal was greater than 120 CPS, and the charge number was +2 ~ +5. The repeated ion collection exclusion time was set at 18 s.

The mass spectrometry data generated by TripleTOF 5600 was retrieved by ProteinPilot (V4.5), and the database retrieval algorithm was Paragon. The database used for retrieval is the reference database of the human protein groups in UniProt. Proteins were screened using the unused ≥ 1.3 standards.

### 2.8. Mouse Model of Endometriosis

The Ethics Committee of Ningbo Women and Children's Hospital approved all animal experiments. All experimental mice were reared in a pathogen-free environment with regulated cycles of light/dark (12 h/12 h, 23-25°C) and easy access to standardized food and ad libitum water. Before any experiments, all experimental mice were given a 10-day adaptation period.

Ten female null mice of specific-pathogen-free (SPF) grade (BalB/C, weighing 21 ± 1.1 g, 6 weeks old) were randomly divided into the control and experimental groups, with 5 mice in each group. Mice were injected intraperitoneally with EMs cell suspension (1 × 10^7^/ml) in both groups. After 20 days, the experimental group was given 10 mg/kg and 200 *μ*l of sunitinib-malate diluent by gavage, while the control group was injected the normal saline. Both groups received gavage once two days and three times in total. The mice's weight and health status were recorded on a daily basis. After a week of observation, the mice were killed and dissected to determine the heterotopic transplantation of endometrial tissue on the intestinal wall.

### 2.9. Immunohistochemistry (IHC)

Formalin-fixed paraffin-embedded (FFPF) mouse ectopic endometrial tissue sections were deparaffinized in xylene, rehydrated with alcohol gradient, and rinsed with PBS. After that, endogenous peroxidase activity was suppressed by exposure to 3% hydrogen peroxide for 20 min. Tissue slides were then blocked with goat serum and then incubated overnight at 4°C with the following primary antibodies: anti-VEGFR (2478, 1 : 300, Cell Signaling Technology, Boston, America), anti-YBX1 (4202, 1 : 50, Cell Signaling Technology, Boston, America), and anti-snail (3879, 1 : 200, Cell Signaling Technology, Boston, America), followed by incubation with HRP-labeled secondary antibodies at room temperature for 1 h, with streptavidin-peroxidase for 30 min in order. Then, sections were visualized by adding DAB (3,3′-diaminobenzidine) substrate, counterstained with hematoxylin, and mounted for observation under the microscope. The presence of yellow staining in the cytoplasm was regarded as a positive expression under the microscope.

### 2.10. Statistical Analysis

The statistical analysis was performed using GraphPad Prism version 6.0 (GraphPad) and SPSS software (version 20.0; IBM Corp., Armonk, NY, USA). Comparisons between two group experiments were performed using a two-tailed Student's *t*-test. The qualification of results was presented as mean ± standard deviation. *P* < 0.05 was considered statistically significant.

## 3. Results

### 3.1. Sunitinib Reduces the Migration of Ectopic Endometriosis Cells

**Figure 1:**
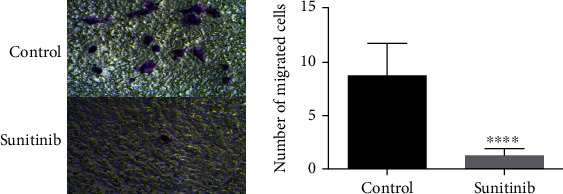
Sunitinib reduced cell migration. The cell number of migration of ectopic endometrial cells in each group. ^∗∗∗∗^*P* < 0.001, and the date in the figure was expressed by mean ± SD. Comparison between two groups was analyzed by *t*-test. The experiment was repeated three times independently (100x).

To observe the effect of sunitinib on the migration ability of ectopic endometrial cells, we first identified endometriotic cells by immunofluorescence (details in SFig. 1). Then, we conducted a transwell experiment. The results showed that the cell number of migration in the control group was higher than that in the sunitinib group, and it was showing a stronger migration ability (Figure 1). Statistical analysis showed that the cell number of migration in the control group was 8.750 ± 0.8627, *N* = 12, while in the sunitinib group was 1.250 ± 0.2176, *N* = 12, showing a statistical meaning (*P* < 0.001). The result testified that sunitinib reduced cell migration.

### 3.2. Sunitinib Inhibited the Expression of Snail

**Figure 2:**
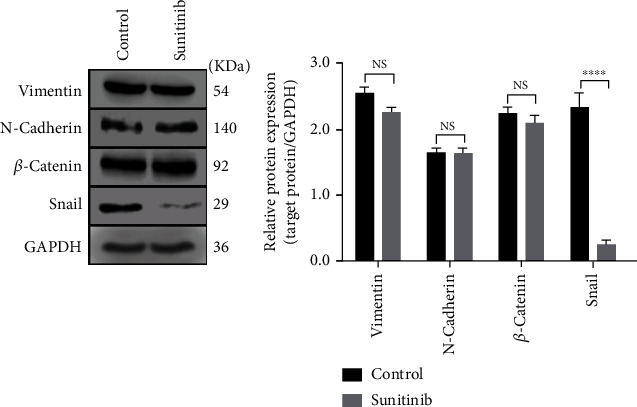
Sunitinib inhibited the expression of snail. Western blot analysis was carried out to detect the protein expression of snail, vimentin, N-cadherin, and *β*-catenin in ectopic cells of two groups and normalized to GAPDH. The expression of vimentin, N-cadherin, and *β*-catenin between two groups was no statistical significance (*P* > 0.5), but the expression of snail decreased significantly (^∗∗∗∗^*P* < 0.0001). The date in the figure was expressed by mean ± SEM, and comparison between two groups was analyzed by *t*-test. The experiment was repeated three times independently. NS: no significance.

Due to the close relationship between migration and EMT, we detected all EMT-related proteins [15], including snail, vimentin, N-cadherin, *β*-catenin, slug, and ZO-1, ZEB1, and claudin-1 in the two groups. However, only snail, vimentin, N-cadherin, and *β*-catenin were checked up protein bands. After comparison with the control group, only the expression of snail decreased, while other proteins remained unchanged in the experimental group (Figure 2). As a result, we hypothesised that sunitinib could reduce the ability of ectopic cells to migrate by inhibiting snail expression via a specific signalling pathway.

### 3.3. Protein Signaling Pathway Prediction

**Figure 3:**
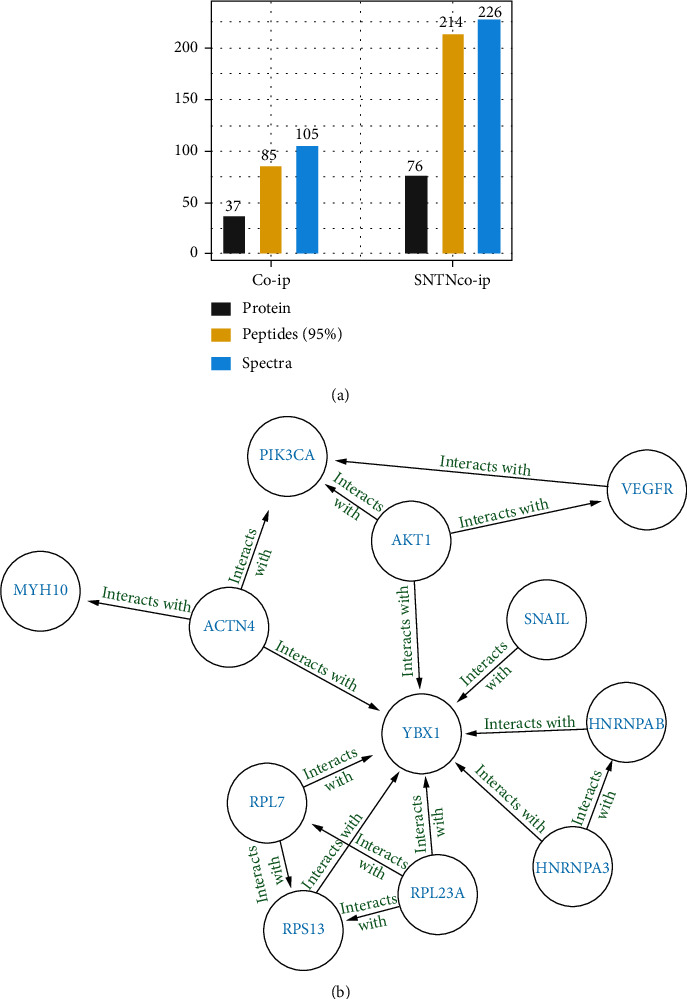
The sample was detected by protein spectrum, and proteins (unused ≥ 1.3) were screened followed by calculation. (a) Peptides (95%), confidence ≥ 95%, and the number of repeated proteins were removed; spectra, the number of secondary mass spectrograms corresponding to the protein. (b) The related signal pathway was analysed by STRING analysis.

To further investigate the mechanism by which sunitinib inhibits snail expression, we used coimmunoprecipitation (Co-IP) technology to precipitate snail-related peptides and analyzed them by protein spectrum. In the Co-IP protein spectrum results, we obtained proteins whose unused ≥1.3. The control group had 37 types of proteins and 85 types of peptides, while the experiment group had 76 types of proteins and 214 types of peptides (Figure 3(a)).

The score (unused), molecular weight (Mw (kD)), and proportion of peptides with 95% confidence in protein sequence (% Cov (95)) between the control group and the experiment group were compared (Table 1). Snail-related proteins were chosen for sunitinib treatment based on bioinformatic analysis. Then, we forecast that p-VEGFR-PI3K-AKT-YBX1-Snail might be the signaling pathway in ectopic endometrium cells after STRING analysis (Figure 3(b)). This indicated that sunitinib inhibited the target VEGFR, and then, proteins of the pathway were reversed, reducing the expression of snail and ultimately inhibiting migration ability.

### 3.4. Sunitinib Regulated the P-VEGFR-PI3K-AKT-YBX1-Snail Pathway and Caused the Migration of Ectopic Endometrial Cells

**Figure 4:**
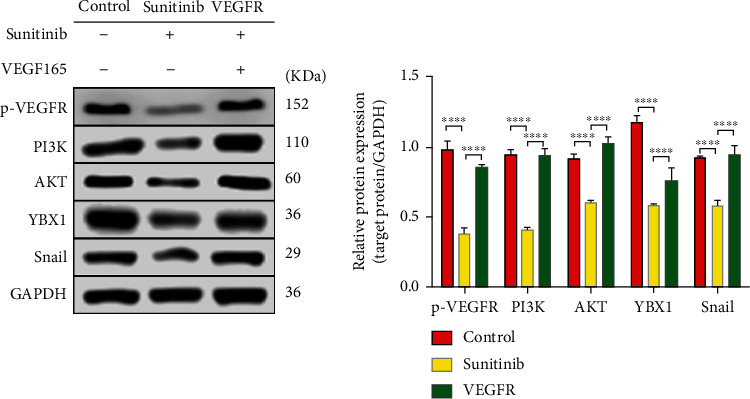
Compared with the control group, proteins were downregulated in the sunitinib group. Compared with the sunitinib group, a part of proteins were upregulated. WB was used to detect the protein expression of p-VEGFR, PI3K, AKT, YBX1, and snail of three groups and normalized to GAPDH. Compared with control group, above proteins were all downregulated in the sunitinib group: p-VEGFR, PI3K, AKT, YBX1, and snail, ^∗∗∗∗^*P* < 0.0001. The VEGFR group was compared with the sunitinib group: p-VEGFR, PI3K, AKT, and snail, ^∗∗∗∗^*P* < 0.0001; YBX1, ^∗∗∗^*P* < 0.001. The data in the figure were expressed by mean ± SD. Comparison between two groups was analyzed by *t*-test, and the experiment was repeated three times independently.

To verify that sunitinib inhibited the expression of snail through the p-VEGFR-PI3K-AKT-YBX1-Snail pathway and thus inhibited the migration of ectopic cells, the experimental group was added with vascular endothelial growth factor 165 (VEGF165). After culture for 24 hours, Western blot was used to test the proteins associated with this signaling pathway in the control group, sunitinib group, and VEGFR group. Compared with the control group, the above pathway proteins in the sunitinib group were all downregulated, which proved that sunitinib could inhibit the pathway. Comparing the VEGFR group with the sunitinb group, p-VEGFR, PI3K, AKT, YBX1, and snail were upregulated in the VEGFR group (Figure 4). So we confirmed the existence of this pathway.

### 3.5. Sunitinib Reduced the Number of Heterotopic Foci and Inhibited the Expression of Snail by In Vivo Experiment

**Figure 5:**
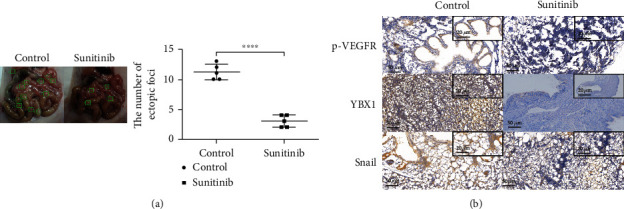
After intragastric administration of sunitinib to mice with EMs, the number of ectopic focus reduced and the expression of p-VEGFR, YBX1, and snail was inhibited. (a) The number of ectopic focus in the small intestine of mice. *t* = 11.16, ^∗∗∗∗^*P* < 0.0001. (b) Immunochemistry was performed to detect the positive rate of p-VEGFR, YBX1, and snail in the control group and sunitinib group (200x, 400x).

For the model mice of EMs, mice in the experimental group were given 10 mg/kg and 200 *μ*l solitinib-malate diluent by gavage, while mice in the control group were given the same amount of normal saline in the same way. The number of ectopic foci was calculated after the mice were killed at the end of the experiment. It was found that the mean number of ectopic focus in the control group was 11.200 ± 0.5831, *n* = 5, while the mean number of ectopic focus in the experiment group was 3.000 ± 0.4472, *N* = 5, *P* < 0.0001 (Figure 5(a)). Therefore, we could conclude that sunitinib reduced the colonization of ectopic focus in the small intestine of mice. To further verify the protein signaling pathway, we conducted IHC experiments. By immunochemistry, we found that the positive rate of p-VEGFR, YBX1, and snail in the sunitinib group were reduced, in line with our expectations, proving that the pathway could be realized in vivo (Figure 5(b)).

## 4. Discussion

At present, epithelial-mesenchymal transition (EMT) is associated with tumor escape, migration, and antiapoptotic ability in cancer stem cells (CSCs) [7]. We also found that some studies have proved that the EMT degree and migration ability of ectopic endometrium is higher than that of normal endometrium [5]. When sunitinib was given to ectopic endometrial cells in this study, we saw a decrease in migration. Therefore, our team examined EMT-related proteins in ectopic endometrium cells with sunitinib treatment and found that the expression of snail reduced; in contrast, the expression of other proteins was not altered. To explain our result, we consulted that, in current studies, most researchers think that the capacity to migrate is linked to the expression of the E-cadherin protein; however, there are opposing perspectives on the biological process of E-cadherin in EMs. Some studies found no difference between ectopic and normal endometrium [16, 17], while others found that the expression was reduced in the ectopic endometrium [18–20]. Our team screened a snail protein that was linked to tumor migration and invasion through a series of experiments. Furthermore, ectopic endometrium cells showed a high level of expression of the snail.

Sunitinib, a multitargeted drug that inhibits VEGFR tyrosine kinase (TK) and other kinases, plays a critical role in tumor growth and metastasis [21]. It is also well known that sunitinib inhibits PI3K/AKT signaling pathways in cancer cell lines and has direct antitumor activity [22]. As a result of the sunitinib treatment, we discovered that the expression of p-VEGFR, AKT, and PI3K was reduced, as well as the ability of ectopic endometrium to migrate, which was in line with our expectations. After that, we detected YBX1 by Co-IP and protein spectrum detection. Through bioinformatics analysis, we considered that sunitinib inhibited VEGFR, which led to the decrease of YBX1 in the ectopic cells with the involvement of the PI3K/AKT signaling pathway [23], finally inhibiting the expression of the snail. And this might be one of the reasons for the decrease in the migration ability of ectopic endometrium cells. Moreover, we activated the target VEGFR by VEGF165 to reverse sunitinib's inhibition of the pathway. As expected, p-VEGFR, PI3K, AKT, YBX1, and snail were upregulated.

In order to deeply validate this result, our team found that sunitinib inhibited the migration of ectopic cells in the model mice of EMs. Similarly, H. Pala et al. found that sunitinib seemed to be effective in the prevention of ectopic foci formation and inhibition of adhesion ability in the rat model of EMs [14], but they did not make an in-depth study of the mechanism. In contrast, we not only focused on in vitro experiments but also demonstrated a sunitinib signaling pathway in vivo.

Sunitinib inhibited the migration of ectopic stromal cells with the involvement of the p-VEGFR-PI3K-AKT-YBX1-Snail signaling pathway; however, it had an obvious side effect on nude mice (the mice lost significant weight during the experiment), and the data was not shown [24, 25]. As a result, future research could reform the drug delivery method, such as wrapping sunitinib in special nanomaterials to reduce side effects and enable sunitinib to kill ectopic endometrium cells with precision. Despite the small population analyzed, sunitinib inhibited the migration of ectopic stromal cells. However, the small sample size is a shortcoming of this study. Subsequently, we will analyze more population in order to clarify drug efficacy and the mechanism of sunitinib.

## 5. Conclusions

We confirmed that sunitinib reduced the migration of ectopic endometrium cells with the involvement of p-VEGFR-PI3K-AKT-YBX1-Snail signaling pathway by both in vitro and in vivo experiments.

## Figures and Tables

**Table 1:** The main different proteins between control_co-ip group and sunitinib_co-ip group.

Accession	Gene	Gene name	Mw (kD)	Unused_co-ip	Unused_SNTNco-ip	%Cov (95)
D6R9P3	HNRNPAB	Heterogeneous nuclear ribonucleoprotein A/B	30.303	4	NA	13.21
P67809	YBX1	Nuclease-sensitive element-binding protein 1	35.924	2	NA	8.95
P62750	RPL23A	60S ribosomal protein L23a	17.695	1.65	NA	8.33
P02042	HBD	Hemoglobin subunit delta	16.055	2	NA	6.80
P62277	RPS13	40S ribosomal protein S13	17.222	1.44	NA	4.64
P18124	RPL7	60S ribosomal protein L7	29.226	1.52	NA	4.43
P07477	PRSS1	Trypsin-1	26.558	2	NA	4.05
P51991	HNRNPA3	Heterogeneous nuclear ribonucleoprotein A3	39.595	2	NA	3.97
P35580	MYH10	Myosin-10	228.999	2.9	NA	1.87
O14983	ATP2A1	Sarcoplasmic/endoplasmic reticulum calcium ATPase 1	110.252	2	NA	1.50
O43707	ACTN4	Alpha-actinin-4	104.854	1.77	NA	1.32

Mw: molecular weight; unused: protein score; %Cov (95): the proportion of peptide with confidence ≥ 95% in the protein sequence. All protein spectrum will be uploaded with supporting files.

## Data Availability

The data that support the findings of this study are available from the corresponding author upon reasonable request.

## Supplementary Materials

Supplementary 1 Supplementary Table 1: detailed clinical information for 3 cases of EMs and 3 cases without EMs.

Supplementary 2 Supplementary Figure 1: sunitinib reduced cell migration. The cell number of migration of ectopic endometrial cells in each group. ^∗∗∗∗^*P* < 0.001, and the date in the figure was expressed by mean ± SD. Comparison between two groups was analyzed by *t*-test. The experiment was repeated three times independently (100x). Supplementary Figure 2: sunitinib inhibited the expression of snail. Western blot analysis was carried out to detect the protein expression of snail, vimentin, N-cadherin, and *β*-catenin in ectopic cells of two groups and normalized to GAPDH. The expression of vimentin, N-cadherin, and *β*-catenin between two groups was no statistical significance (*P* > 0.5), but the expression of snail decreased significantly (^∗∗∗∗^*P* < 0.0001). The date in the figure was expressed by mean ± SEM and comparison between two groups was analyzed by *t*-test. The experiment was repeated three times independently. NS: no significance. Supplementary Figure 3: the sample was detected by protein spectrum, and proteins (unused ≥ 1.3) were screened followed by calculation. (A) Peptides (95%), confidence ≥ 95%, and the number of repeated proteins were removed; spectra: the number of secondary mass spectrograms corresponding to the protein. (B) The related signal pathway was analysed by STRING analysis. Supplementary Figure 4: compared with the control group, proteins were downregulated in the sunitinib group; compared with the sunitinib group, a part of proteins were upregulation. WB was used to detect the protein expression of p-VEGFR, PI3K, AKT, YBX1, and snail of three groups and normalized to GAPDH. Compared with the control group, above proteins were all downregulated in the sunitinib group: p-VEGFR, PI3K, AKT, YBX1, and snail, ^∗∗∗∗^*P* < 0.0001. The VEGFR group was compared with the sunitinib group: p-VEGFR, PI3K, AKT, and snail, ^∗∗∗∗^*P* < 0.0001; YBX1, ^∗∗∗^*P* < 0.001. The data in the figure were expressed by mean ± SD. Comparison between two groups was analyzed by *t*-test, and the experiment was repeated three times independently. Supplementary Figure 5: after intragastric administration of sunitinib to mice with EMs, the number of ectopic focus reduced, and the expression of p-VEGFR, YBX1, and snail was inhibited. (A) The number of ectopic focus in the small intestine of mice. *t* = 11.16, ^∗∗∗∗^*P* < 0.0001. (B) Immunochemistry was performed to detect the positive rate of p-VEGFR, YBX1, and snail in the control group and sunitinib group (200x, 400x).
