# Supplementary material for: Sunitinib Reduced the Migration of Ectopic Endometrial Cells via p-VEGFR-PI3K-AKT-YBX1-Snail Signaling Pathway
Source: Anal Cell Pathol (Amst). 2022 Jun 30;2022:6042518. doi: 10.1155/2022/6042518 (PMC9274230; doi:10.1155/2022/6042518)
Supplement: Supplementary 1 — Supplementary Table 1: detailed clinical information for 3 cases of EMs and 3 cases without EMs. [file 6042518.f1.docx]

**Supplementary Table1** | Subject’s Characteristics in women with and without endometriosis

| **Parameters** | **Endometriosis (n=3）** | **No Endometriosis (n=3)** | **P value** |
| --- | --- | --- | --- |
| **Age** | 33.67±1.2 | 34.33±2.0 | 0.7913 |
| **Dysmenorrhea** | 3(100%) | 2(66.67%) |  |
| **Menstrual cycle phase** |  |  |  |
| Proliferative | 2(66.67%) | 3(100.00%) |  |
| Secretory | 1(33.33%) | 0(0.00%) |  |
| **Size of Cyst(cm^3^)** |  |  |  |
| <64cm^3^ | 1(33.33%) |  |  |
| <216cm^3^,≥64cm^3^ | 1(33.33%) |  |  |
| ≥216cm^3^ | 1(33.33%) |  |  |
