# Supplementary material for: Sunitinib Reduced the Migration of Ectopic Endometrial Cells via p-VEGFR-PI3K-AKT-YBX1-Snail Signaling Pathway
Source: Anal Cell Pathol (Amst). 2022 Jun 30;2022:6042518. doi: 10.1155/2022/6042518 (PMC9274230; doi:10.1155/2022/6042518)
Supplement: Supplementary 2 — Supplementary Figure 1: sunitinib reduced cell migration. The cell number of migration of ectopic endometrial cells in each group. ∗∗∗∗P < 0.001, and the date in the figure was expressed by mean ± SD. Comparison between two groups was analyzed by t-test. The experiment was repeated three times independently (100x). Supplementary Figure 2: sunitinib inhibited the expression of snail. Western blot analysis was carried out to detect the protein expression of snail, vimentin, N-cadherin, and β-catenin in ectopic cells of two groups and normalized to GAPDH. The expression of vimentin, N-cadherin, and β-catenin between two groups was no statistical significance (P > 0.5), but the expression of snail decreased significantly (∗∗∗∗P < 0.0001). The date in the figure was expressed by mean ± SEM and comparison between two groups was analyzed by t-test. The experiment was repeated three times independently. NS: no significance. Supplementary Figure 3: the sample was detected by protein spectrum, and proteins (unused ≥ 1.3) were screened followed by calculation. (A) Peptides (95%), confidence ≥ 95%, and the number of repeated proteins were removed; spectra: the number of secondary mass spectrograms corresponding to the protein. (B) The related signal pathway was analysed by STRING analysis. Supplementary Figure 4: compared with the control group, proteins were downregulated in the sunitinib group; compared with the sunitinib group, a part of proteins were upregulation. WB was used to detect the protein expression of p-VEGFR, PI3K, AKT, YBX1, and snail of three groups and normalized to GAPDH. Compared with the control group, above proteins were all downregulated in the sunitinib group: p-VEGFR, PI3K, AKT, YBX1, and snail, ∗∗∗∗P < 0.0001. The VEGFR group was compared with the sunitinib group: p-VEGFR, PI3K, AKT, and snail, ∗∗∗∗P < 0.0001; YBX1, ∗∗∗P < 0.001. The data in the figure were expressed by mean ± SD. Comparison between two groups was analyzed by t-test, and the experiment [file 6042518.f2.doc]

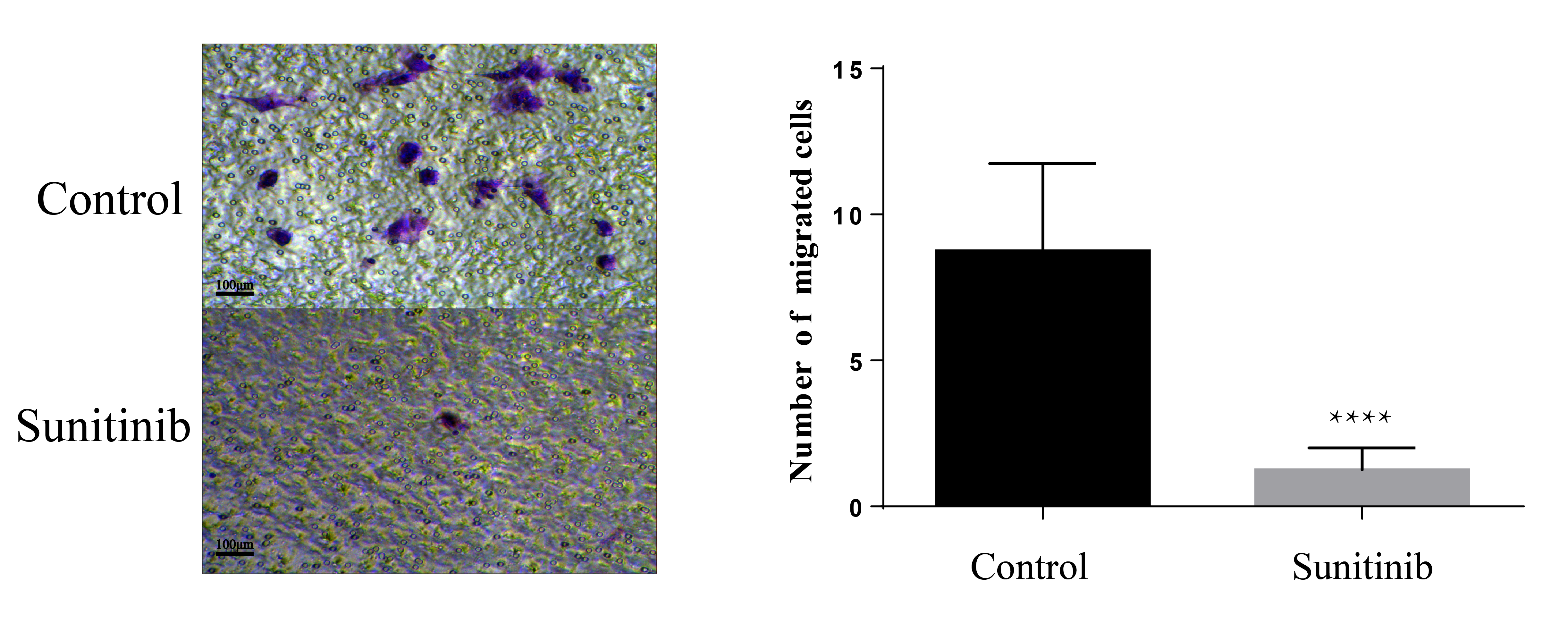


**Supplementary Figure 1:** Sunitinib reduced cell migration. The cell number of migration of ectopic endometrial cells in each group. ****P<0.001, and the date in the figure was expressed by Mean±SD. Comparison between two groups was analyzed by t-test. The experiment was repeated three times independently (100×).

**
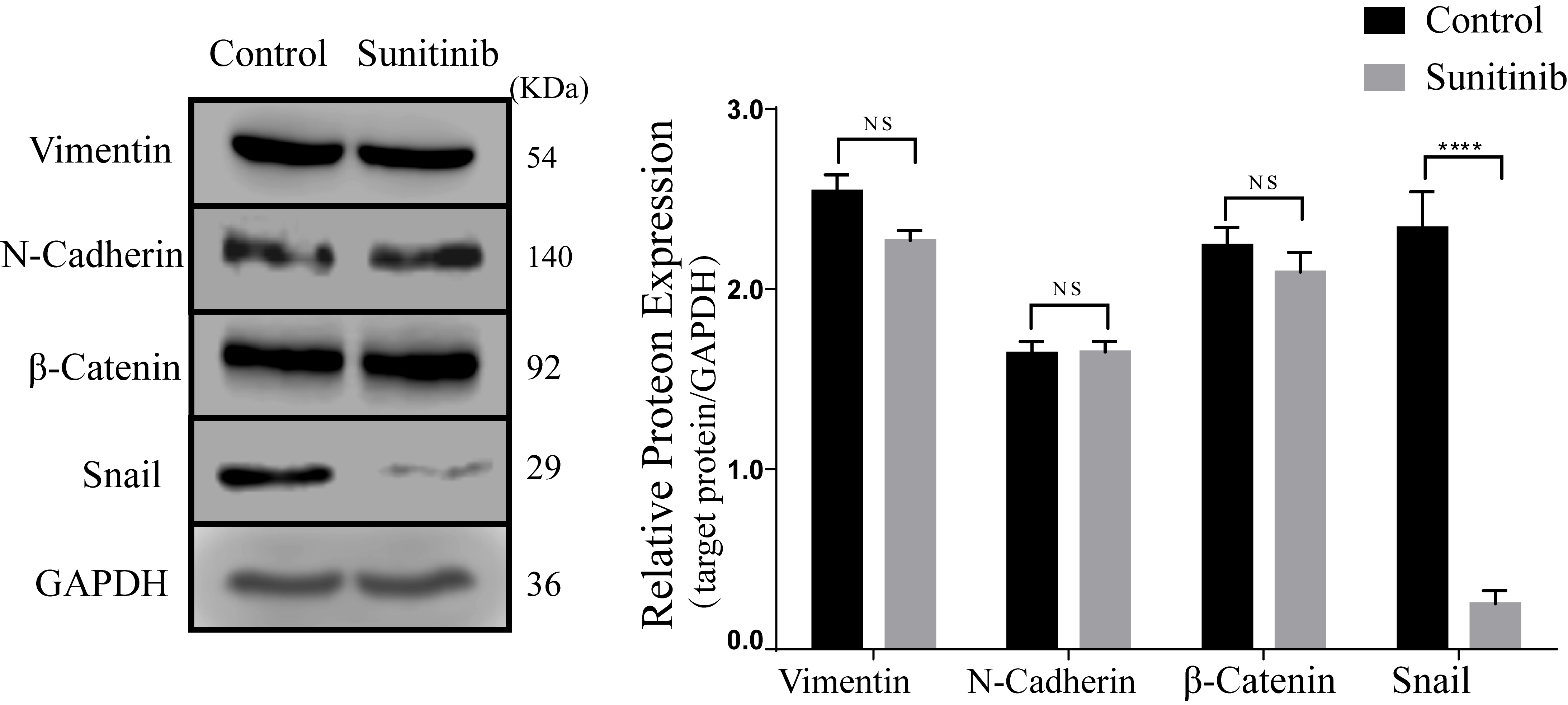
**

**Supplementary Figure 2:** Sunitinib inhibited the expression of snail. Western blot analysis was carried out to detect the protein expression of snail, Vimentin, N-Cadherin, and β-Catenin in ectopic cells of two groups and normalized to GAPDH. The expression of Vimentin, N-Cadherin, β-Catenin between two groups were no statistical significance (P>0.5), but the expression of snail decreased significantly (****P<0.0001). The date in the figure was expressed by mean±SEM and comparison between two groups was analyzed by t-test. The experiment was repeated three times independently. NS, no significance.

**
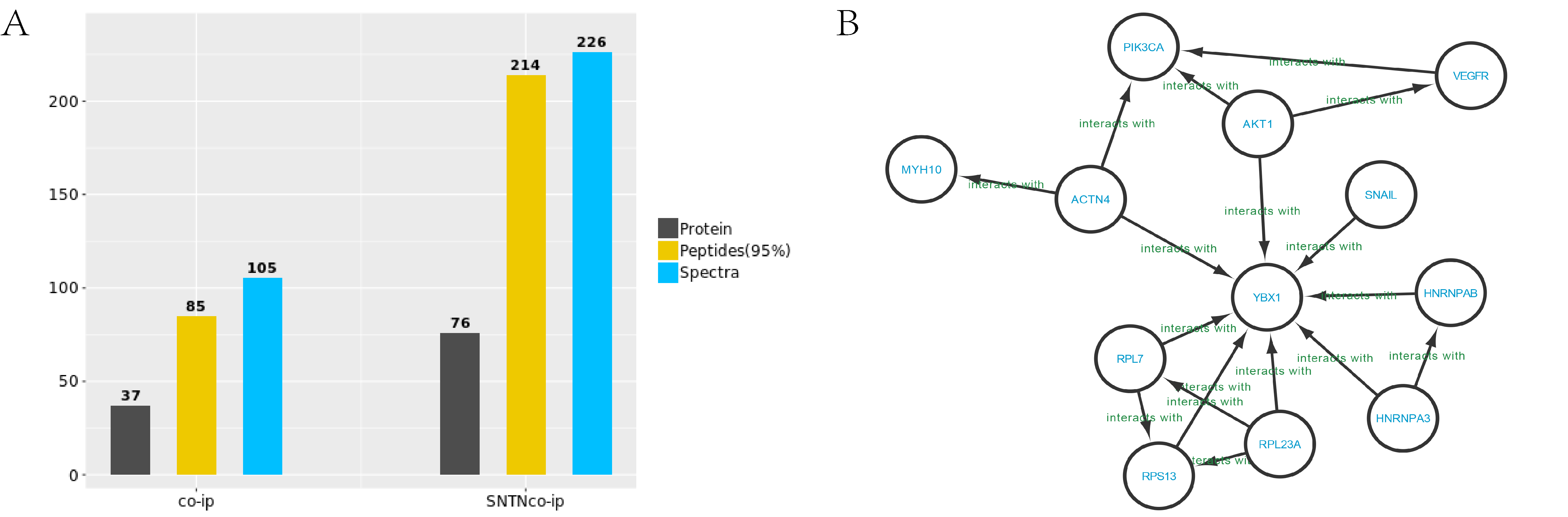
**

**Supplementary Figure 3:** The sample was detected by protein spectrum, and proteins (unused≥1.3) were screened followed by calculation. (A) Peptides (95%), confidence ≥95%, and the number of repeated proteins were removed; Spectra, the number of secondary mass spectrograms corresponding to the protein. (B) The related signal pathway was analysed by String analysis.

**
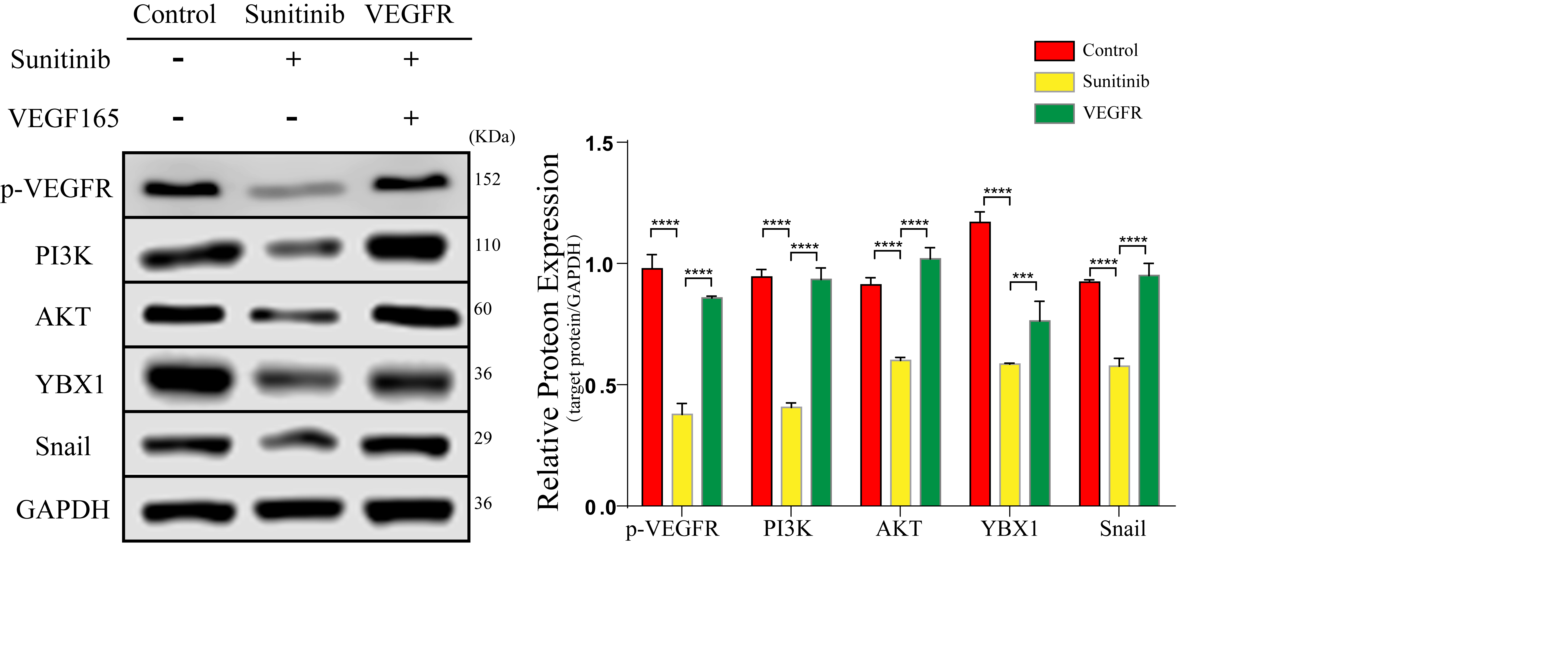
**

**Supplementary Figure 4:** Compared with the control group, proteins were down-regulated in the sunitinib group; Compared with sunitinib group, a part of proteins were up-regulation. WB was used to detect the protein expression of p-VEGFR, PI3K, AKT, YBX1 and snail of three groups and normalized to GAPDH. Compared with control group, above proteins were all down-regulated in the sunitinib group: p-VEGFR, PI3K, AKT, YBX1 and snail, ****P<0.0001. VEGFR group were compared with sunitinib group: p-VEGFR, PI3K, AKT and snail, ****P<0.0001; YBX1, ***P<0.001. The data in the figure were expressed by mean ± SD. Comparison between two groups was analyzed by t-test and the experiment was repeated three times independently.

**Supplementary Figure 5:** After intragastric administration of sunitinib to mice with EMs, the number of ectopic focus reduced and the expression of p-VEGFR, YBX1, snail was inhibited. (A) The number of ectopic focus in the [small intestine](http://dict.youdao.com/w/small intestine/" \l "keyfrom=E2Ctranslation) of mice. t=11.16, ****P<0.0001. (B) Immunochemistry was performed to detect the positive rate of p-VEGFR, YBX1 and snail in control group and sunitinib group (200×, 400×).
